# Supplementary material for: Nanopore sequencing of SARS-CoV-2: Comparison of short and long PCR-tiling amplicon protocols
Source: PLoS One. 2021 Oct 29;16(10):e0259277. doi: 10.1371/journal.pone.0259277 (PMC8555800; doi:10.1371/journal.pone.0259277)
Supplement: S1 Table — (PDF) [file pone.0259277.s001.pdf]

S1 Table. Overview of the SARS-CoV-2 samples sequenced in this study.

Batch	Sample ID	Sample	Sample	RT-qPCR	Barcode	GISAID ID	Pangolin lineage
UKBA-2	UKBA-201/2020	2020-07-09	swab	21.36	01	EPI_ISL_577734	B.1.1.266
	UKBA-202/2020	2020-03-30	swab	31.06	02	not submitted	n/a
	UKBA-203/2020	2020-04-06	swab	19.34	03	EPI_ISL_577735	B.1.1
	UKBA-204/2020	2020-04-06	swab	22.15	04	EPI_ISL_577736	B.1.1
	UKBA-205/2020	2020-04-13	swab	18.40	05	EPI_ISL_577737	B.1.1
	UKBA-206/2020	2020-04-14	swab	20.57	06	not submitted	n/a
	UKBA-207/2020	2020-04-29	swab	30.80	07	EPI_ISL_577738	B.1.1
	UKBA-208/2020	2020-05-06	swab	32.03	08	EPI_ISL_577739	B.1.1
	UKBA-209/2020	2020-06-30	swab	22.09	09	EPI_ISL_577740	B.1.1.70
	UKBA-210/2020	2020-06-30	swab	18.46	10	EPI_ISL_577741	B.1.131
	UKBA-211/2020	2020-07-07	swab	31.49	11	not submitted	n/a
	UKBA-212/2020	2020-03-31	swab	24.36	12	EPI_ISL_577742	B.1.1
UKBA-3	UKBA-313/2020	2020-09-10	swab	25.58	13	EPI_ISL_583481	B.1.1.70
	UKBA-314/2020	2020-09-10	swab	25.37	14	EPI_ISL_583482	B.1.160
	UKBA-315/2020	2020-09-10	swab	23.12	15	EPI_ISL_583483	B.1.160
	UKBA-316/2020	2020-09-10	swab	18.05	16	EPI_ISL_583484	B.1.160
	UKBA-317/2020	2020-09-16	swab	17.87	17	EPI_ISL_583485	B.1.160
	UKBA-318/2020	2020-09-16	swab	24.28	18	EPI_ISL_583486	B.1.1.170
	UKBA-319/2020	2020-09-16	swab	26.67	19	EPI_ISL_583487	B.1.1.70
	UKBA-320/2020	2020-09-16	swab	20.50	20	EPI_ISL_717975	B.1.1
	UKBA-321/2020	2020-09-16	swab	18.11	21	EPI_ISL_583488	B.1.416.1
	UKBA-322/2020	2020-09-16	swab	16.64	22	EPI_ISL_583489	B.1.1.70
UKBA-4	UKBA-401/2020	2020-11-22	swab	21.03	01	EPI_ISL_779403	B.1.1.163
	UKBA-402/2020	2020-11-22	swab	17.90	02	EPI_ISL_718250	B.1.160
	UKBA-403/2020	2020-11-27	swab	20.22	03	EPI_ISL_718251	B.1.258
	UKBA-404/2020	2020-11-30	swab	16.50	04	EPI_ISL_718252	B.1.258
	UKBA-405/2020	2020-11-30	swab	17.24	05	EPI_ISL_718253	B.1.258
	UKBA-406/2020	2020-11-30	swab	14.54	06	EPI_ISL_718254	B.1.160
	UKBA-407/2020	2020-11-30	swab	17.41	07	EPI_ISL_718255	B.1.160
	UKBA-408/2020	2020-11-30	swab	17.72	08	EPI_ISL_718256	B.1.160
	UKBA-409/2020	2020-11-19	swab	22.72	09	EPI_ISL_718257	B.1.1.170
	UKBA-410/2020	2020-11-19	swab	17.62	10	EPI_ISL_718258	B.1.527
	UKBA-411/2020	2020-11-13	swab	18.89	11	EPI_ISL_718259	B.1.1.243
	UKBA-412/2020	2020-11-12	swab	19.16	12	EPI_ISL_718260	B.1.1.170
UKBA-6	UKBA-601/2020	2020-11-12	swab	17.54	01	EPI_ISL_788979	B.1.160
	UKBA-602/2020	2020-11-12	swab	17.95	02	EPI_ISL_788980	B.1.527
	UKBA-603/2020	2020-11-12	swab	20.21	03	EPI_ISL_788981	B.1.527
	UKBA-604/2020	2020-11-12	swab	20.05	04	EPI_ISL_788982	B.1.258
	UKBA-605/2020	2020-11-23	swab	24.00	05	EPI_ISL_791990	B.1.1.163
	UKBA-606/2020	2020-11-27	swab	24.17	06	EPI_ISL_788983	B.1.160
	UKBA-607/2020	2020-11-30	swab	20.65	07	EPI_ISL_788984	B.1.160
	UKBA-608/2020	2020-11-23	swab	23.30	08	EPI_ISL_788985	B.1.258
	UKBA-609/2020	2020-11-30	swab	20.37	09	EPI_ISL_788986	B.1.160
	UKBA-610/2020	2020-11-30	swab	21.59	10	EPI_ISL_788987	B.1.160
	UKBA-611/2020	2020-11-30	swab	25.67	11	EPI_ISL_788988	B.1.160
UKBA-10	UKBA-1001/2020	2020-12-22	swab	15.00	01	EPI_ISL_903980	B.1.258
	UKBA-1002/2020	2020-12-22	swab	20.20	02	EPI_ISL_903981	B.1.1.170
	UKBA-1003/2020	2020-12-22	swab	22.70	03	EPI_ISL_903982	B.1.258
	UKBA-1004/2020	2020-12-22	swab	14.90	04	EPI_ISL_903983	B.1.258
	UKBA-1005/2020	2020-12-22	swab	20.20	05	EPI_ISL_903984	B.1.258
	UKBA-1006/2020	2020-12-22	swab	13.90	06	EPI_ISL_903985	B.1.258
	UKBA-1007/2020	2020-12-22	swab	13.70	07	EPI_ISL_903986	B.1.1.7
	UKBA-1008/2020	2020-12-22	swab	16.10	08	EPI_ISL_903987	B.1.258
	UKBA-1009/2020	2020-12-22	swab	17.70	09	EPI_ISL_903988	B.1.160
	UKBA-1010/2020	2020-12-22	swab	17.30	10	EPI_ISL_903989	B.1.1.7
	UKBA-1011/2020	2020-12-22	swab	21.40	11	EPI_ISL_903990	B.1.1.7
	UKBA-1012/2020	2020-12-22	swab	21.50	12	EPI_ISL_903991	B.1.258
	UKBA-1013/2020	2020-12-22	swab	14.20	13	EPI_ISL_903992	B.1.177
	UKBA-1014/2020	2020-12-22	swab	20.10	14	EPI_ISL_903993	B.1.258
	UKBA-1015/2020	2020-12-22	swab	22.70	15	EPI_ISL_903994	B.1.1.277
	UKBA-1016/2020	2020-12-22	swab	19.90	16	EPI_ISL_903995	B.1.258
	UKBA-1017/2020	2020-12-22	swab	16.70	17	EPI_ISL_903996	B.1.160
	UKBA-1018/2020	2020-12-22	swab	16.40	18	EPI_ISL_903997	B.1.258
	UKBA-1019/2020	2020-12-22	swab	15.90	19	EPI_ISL_903998	B.1.258
	UKBA-1020/2020	2020-12-22	swab	14.70	20	EPI_ISL_903999	B.1.258
	UKBA-1021/2020	2020-12-22	swab	16.90	21	EPI_ISL_904000	B.1.1.7
	UKBA-1022/2020	2020-12-22	swab	14.80	22	EPI_ISL_904001	B.1.221
	UKBA-1023/2020	2020-12-22	swab	18.70	23	EPI_ISL_904002	B.1.258
	UKBA-1024/2020	2020-12-22	swab	22.50	24	EPI_ISL_904003	B.1.258
UKBA-11	UKBA-1101/2021	2021-01-20	swab	19.23	01	EPI_ISL_959643	B.1.1.7
	UKBA-1102/2021	2021-01-20	swab	16.87	02	EPI_ISL_959642	B.1.160
	UKBA-1103/2021	2021-01-20	swab	16.35	03	EPI_ISL_959648	B.1.258
	UKBA-1104/2021	2021-01-20	swab	14.93	04	EPI_ISL_959645	B.1.1.7
	UKBA-1105/2021	2021-01-20	swab	17.68	05	EPI_ISL_959647	B.1.1.170
	UKBA-1106/2021	2021-01-20	swab	18.64	06	EPI_ISL_959646	B.1.1.7
	UKBA-1107/2021	2021-01-20	swab	21.26	07	EPI_ISL_959644	B.1.1.7

	UKBA-1108/2021	2021-01-20	swab	19.74	08	EPI_ISL_959649	B.1.258
	UKBA-1109/2021	2021-01-21	swab	24.25	09	EPI_ISL_959637	B.1.1.7
	UKBA-1110/2021	2021-01-21	swab	21.76	10	EPI_ISL_959638	B.1.1.7
	UKBA-1111/2021	2021-01-21	swab	16.65	11	EPI_ISL_959639	B.1.1.7
	UKBA-1112/2021	2021-01-21	swab	20.69	12	EPI_ISL_959640	B.1.1.7
	UKBA-1113/2021	2021-01-21	swab	22.52	13	EPI_ISL_959641	B.1.1.170
	UKBA-1114/2021	2021-01-28	swab	23.99	14	EPI_ISL_959627	B.1.1.7
	UKBA-1115/2021	2021-01-28	swab	23.14	15	EPI_ISL_959630	B.1.258
	UKBA-1116/2021	2021-01-28	swab	25.20	16	EPI_ISL_959628	B.1.1.7
	UKBA-1117/2021	2021-01-28	swab	23.07	17	EPI_ISL_959626	B.1.1.7
	UKBA-1118/2021	2021-01-28	swab	16.29	18	EPI_ISL_959631	B.1.258
	UKBA-1119/2021	2021-01-28	swab	17.18	19	EPI_ISL_959629	B.1.1.7
	UKBA-1120/2021	2021-01-26	swab	15.78	20	EPI_ISL_959632	B.1.1.7
	UKBA-1121/2021	2021-01-26	swab	16.60	21	EPI_ISL_959633	B.1.1.7
	UKBA-1122/2021	2021-01-26	swab	15.92	22	EPI_ISL_959634	B.1.1.7
	UKBA-1123/2021	2021-01-26	swab	15.92	23	EPI_ISL_959635	B.1.1.7
	UKBA-1124/2021	2021-01-26	swab	16.75	24	EPI_ISL_959636	B.1.258
UKBA-12	UKBA-1201/2021	2021-01-20	swab	17.63	01	EPI_ISL_959623	B.1.258
	UKBA-1202/2021	2021-01-20	swab	17.78	02	EPI_ISL_959624	B.1.1.7
	UKBA-1203/2021	2021-01-20	swab	17.64	03	EPI_ISL_959625	B.1.1.170
	UKBA-1204/2021	2021-01-21	swab	15.27	04	EPI_ISL_959622	B.1.1.7
	UKBA-1205/2021	2021-01-26	swab	16.87	05	EPI_ISL_1234384	B.1.1.7
	UKBA-1206/2021	2021-01-26	swab	15.31	06	EPI_ISL_959621	B.1.1.7
	UKBA-1207/2021	2021-02-02	swab	16.49	07	EPI_ISL_959604	B.1.1.7
	UKBA-1208/2021	2021-02-02	swab	23.46	08	EPI_ISL_959605	B.1.1.7
	UKBA-1209/2021	2021-02-02	swab	13.46	09	EPI_ISL_959606	B.1.1.7
	UKBA-1210/2021	2021-02-02	swab	14.50	10	EPI_ISL_959607	B.1.258
	UKBA-1211/2021	2021-02-02	swab	17.59	11	EPI_ISL_959608	B.1.1.7
	UKBA-1212/2021	2021-02-02	swab	15.51	12	EPI_ISL_959609	B.1.1.7
	UKBA-1213/2021	2021-02-02	swab	21.57	13	EPI_ISL_959610	B.1.1.7
	UKBA-1214/2021	2021-02-02	swab	14.68	14	EPI_ISL_959611	B.1.1.7
	UKBA-1215/2021	2021-02-02	swab	17.21	15	EPI_ISL_959612	B.1.1.7
	UKBA-1216/2021	2021-02-02	swab	13.69	16	EPI_ISL_959613	B.1.1.7
	UKBA-1217/2021	2021-02-02	swab	14.57	17	EPI_ISL_959614	B.1.1.7
	UKBA-1218/2021	2021-02-02	swab	22.37	18	EPI_ISL_959615	B.1.1.7
	UKBA-1219/2021	2021-02-02	swab	15.54	19	EPI_ISL_959616	B.1.1.7
	UKBA-1221/2021	2021-02-02	swab	17.71	21	EPI_ISL_959617	B.1.1.7
	UKBA-1222/2021	2021-02-02	swab	14.06	22	EPI_ISL_959618	B.1.1.7
	UKBA-1223/2021	2021-02-02	swab	24.14	23	EPI_ISL_959619	B.1.258
	UKBA-1224/2021	2021-02-02	swab	17.61	24	EPI_ISL_959620	B.1.258
UKBA-19	UKBA-1901/2021	2021-03-08	swab	19.45	01	EPI_ISL_1299292	B.1.1.7
	UKBA-1902/2021	2021-03-08	swab	23.44	02	EPI_ISL_1299293	B.1.1.7
	UKBA-1903/2021	2021-03-07	swab	23.52	03	EPI_ISL_1299294	B.1.1.7
	UKBA-1904/2021	2021-03-07	swab	18.47	04	EPI_ISL_1299295	B.1.1.7
	UKBA-1905/2021	2021-03-08	swab	16.57	05	EPI_ISL_1299296	B.1.1.7
	UKBA-1906/2021	2021-03-08	swab	21.61	06	EPI_ISL_1299297	B.1.1.7
	UKBA-1907/2021	2021-03-09	swab	21.51	07	EPI_ISL_1299298	B.1.1.7
	UKBA-1908/2021	2021-03-08	swab	18.15	08	EPI_ISL_1299299	B.1.1.7
	UKBA-1909/2021	2021-03-09	swab	22.39	09	EPI_ISL_1299300	B.1.1.7
	UKBA-1910/2021	2021-03-09	swab	22.55	10	EPI_ISL_1299301	B.1.1.7
	UKBA-1911/2021	2021-03-09	swab	14.16	11	EPI_ISL_1299302	B.1.1.7
	UKBA-1912/2021	2021-03-09	swab	24.10	12	EPI_ISL_1299303	B.1.1.7
	UKBA-1913/2021	2021-03-10	swab	15.70	13	EPI_ISL_1299304	B.1.1.7
	UKBA-1914/2021	2021-03-10	swab	19.90	14	EPI_ISL_1299305	B.1.1.7
	UKBA-1915/2021	2021-03-09	swab	22.50	15	EPI_ISL_1299306	B.1.1.7
	UKBA-1916/2021	2021-03-09	swab	25.60	16	EPI_ISL_1299307	B.1.1.7
	UKBA-1917/2021	2021-03-09	swab	18.60	17	EPI_ISL_1299308	B.1.1.7
	UKBA-1918/2021	2021-03-10	swab	22.60	18	EPI_ISL_1299309	B.1.1.7
	UKBA-1919/2021	2021-03-10	swab	19.60	19	EPI_ISL_1299310	B.1.1.7
	UKBA-1920/2021	2021-03-10	swab	23.60	20	EPI_ISL_1299311	B.1.1.7
	UKBA-1921/2021	2021-03-10	swab	20.10	21	EPI_ISL_1299312	B.1.1.7
	UKBA-1922/2021	2021-03-11	swab	24.40	22	EPI_ISL_1299313	B.1.1.7
	UKBA-1923/2021	2021-03-08	gargle	24.32	23	EPI_ISL_1299314	B.1.1.7
	UKBA-1924/2021	2021-03-10	gargle	17.62	24	EPI_ISL_1299315	B.1.1.7
UKBA-21	UKBA-2101/2021	2021-03-17	swab	19.59	01	EPI_ISL_1347634	B.1.1.7
	UKBA-2102/2021	2021-03-17	swab	19.25	02	EPI_ISL_1347635	B.1.1.7
	UKBA-2103/2021	2021-03-17	swab	19.34	03	EPI_ISL_1347636	B.1.1.7
	UKBA-2104/2021	2021-03-17	swab	19.93	04	EPI_ISL_1347637	B.1.1.7
	UKBA-2105/2021	2021-03-17	swab	20.14	05	EPI_ISL_1347638	B.1.1.7
	UKBA-2106/2021	2021-03-16	swab	19.28	06	EPI_ISL_1347639	B.1.1.7
	UKBA-2107/2021	2021-03-16	swab	18.36	07	EPI_ISL_1347640	B.1.1.7
	UKBA-2108/2021	2021-03-16	swab	15.65	08	EPI_ISL_1347641	B.1.1.7
	UKBA-2109/2021	2021-03-18	swab	19.96	09	EPI_ISL_1347642	B.1.1.7
	UKBA-2110/2021	2021-03-18	swab	18.44	10	EPI_ISL_1347643	B.1.1.7
	UKBA-2111/2021	2021-03-19	swab	19.01	11	EPI_ISL_1347644	B.1.1.7
	UKBA-2112/2021	2021-03-19	swab	22.94	12	EPI_ISL_1347645	B.1.1.7

sequencing of three samples shown in grey failed
