# Supplementary figures and images for: Nanopore sequencing of SARS-CoV-2: Comparison of short and long PCR-tiling amplicon protocols
Source: PLoS One. 2021 Oct 29;16(10):e0259277. doi: 10.1371/journal.pone.0259277 (PMC8555800; doi:10.1371/journal.pone.0259277)

S1 Fig

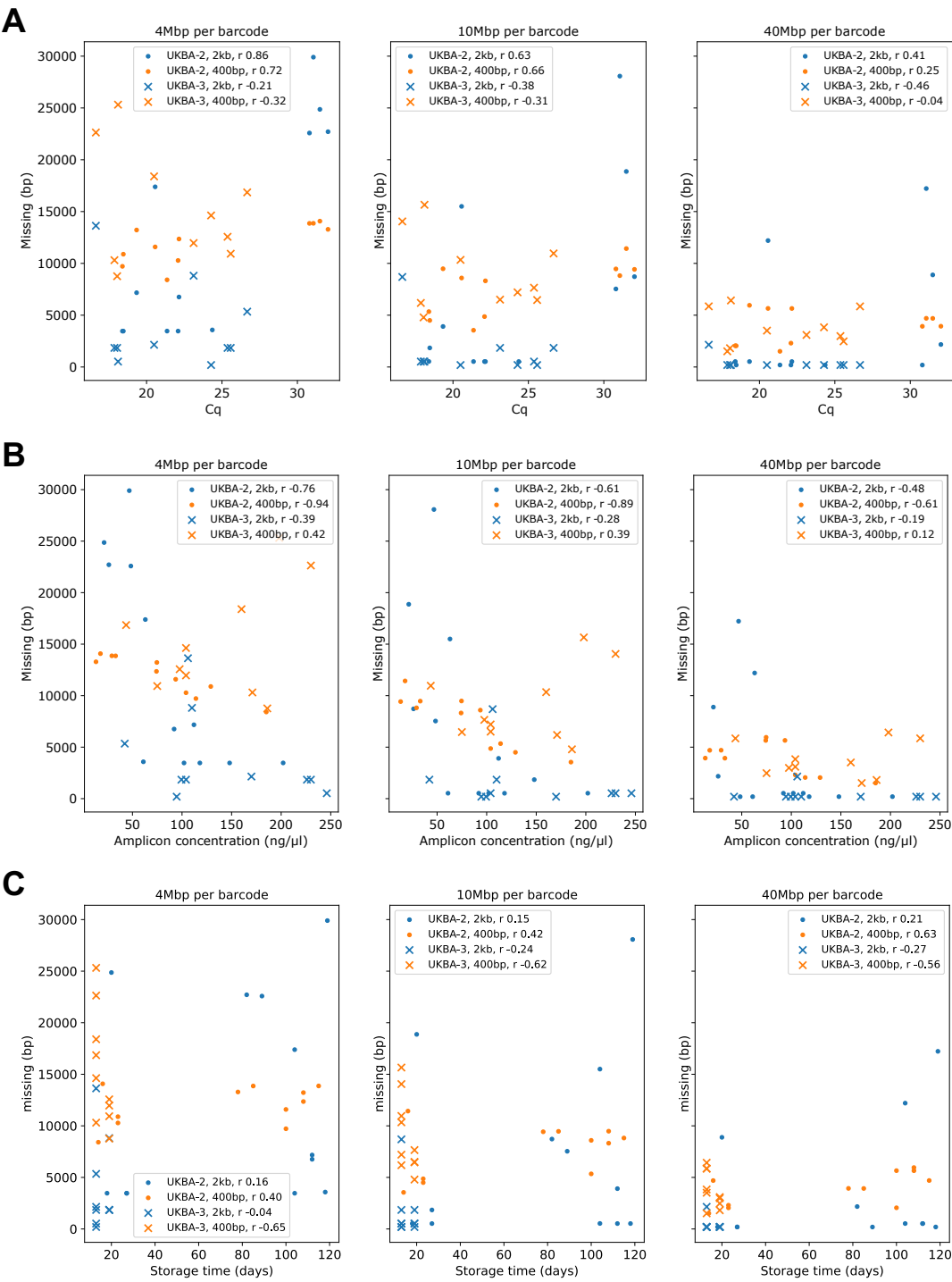

Supplement: S1 Fig — (A) Cq value of the diagnostic RT-qPCR test, (B) DNA concentration after amplification, (C) length of storage of the sample before PCR amplification. Each dot corresponds to one sample, each sub-plot has a different level of sequencing per barcode. (PDF) [file pone.0259277.s002.pdf]

## S2 Fig

**A**

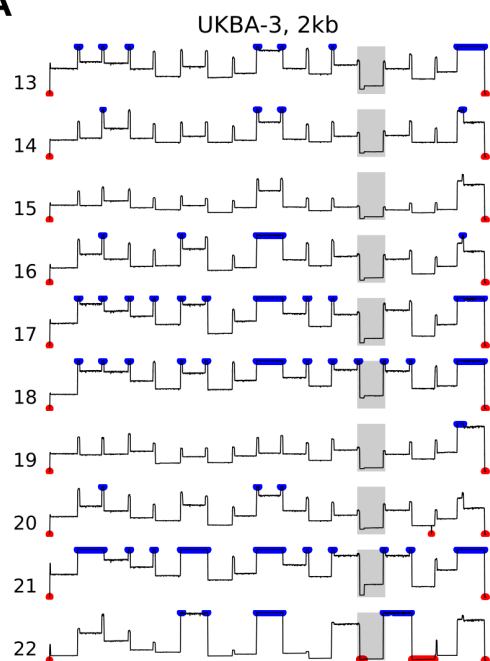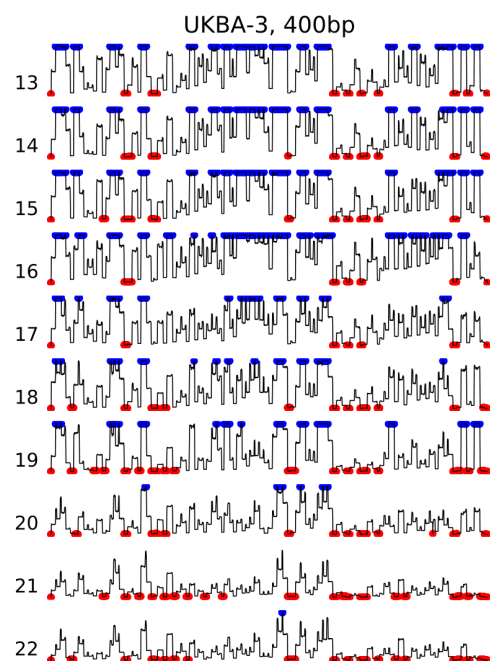

## S2 Fig (continued)

**B**

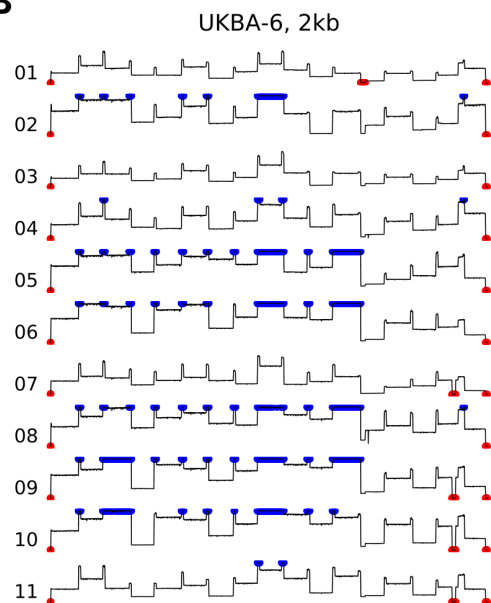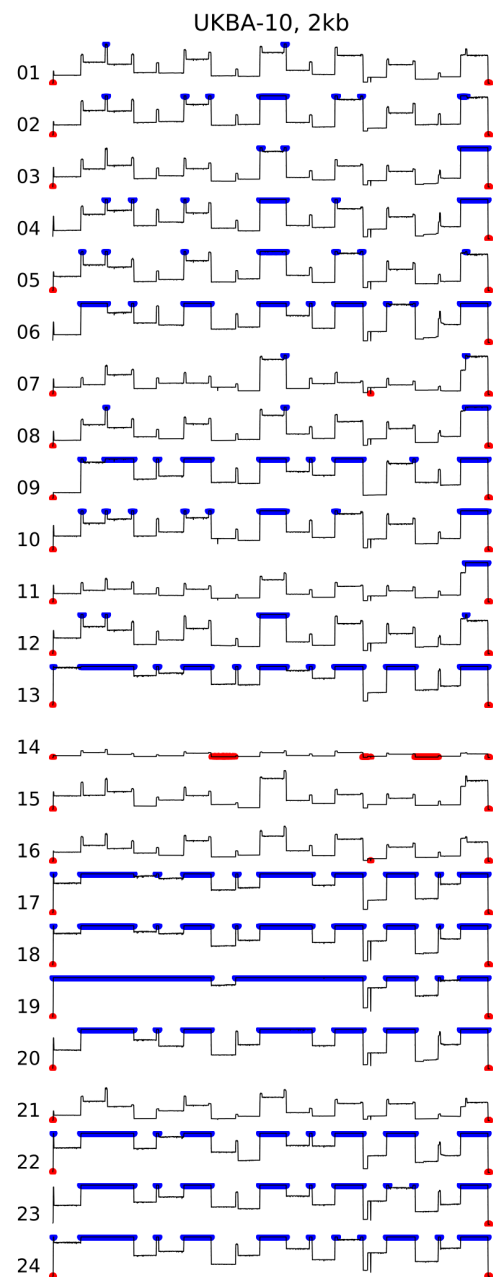

## S2 Fig (continued)

**C**

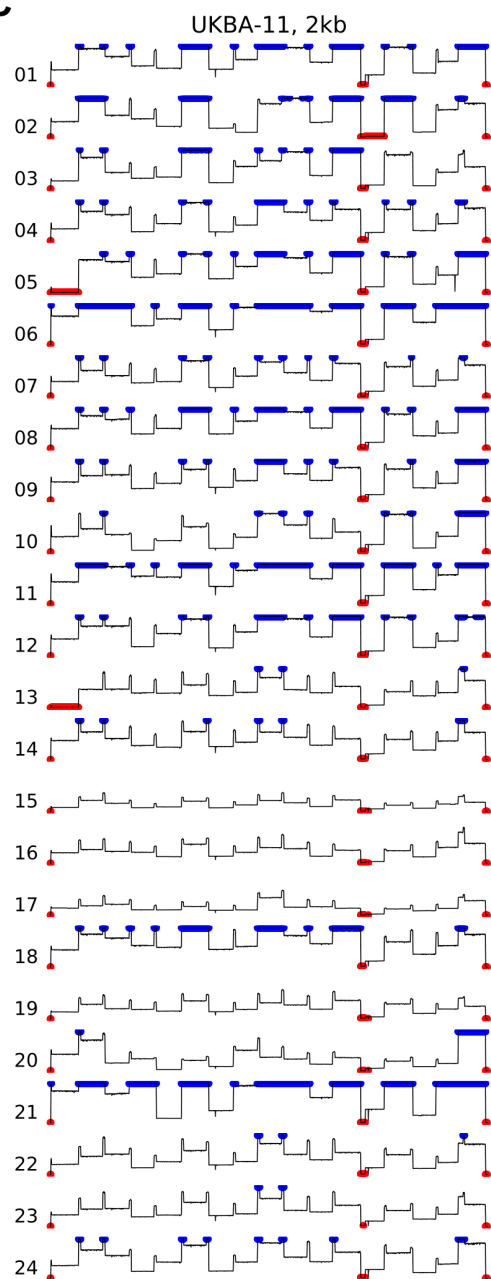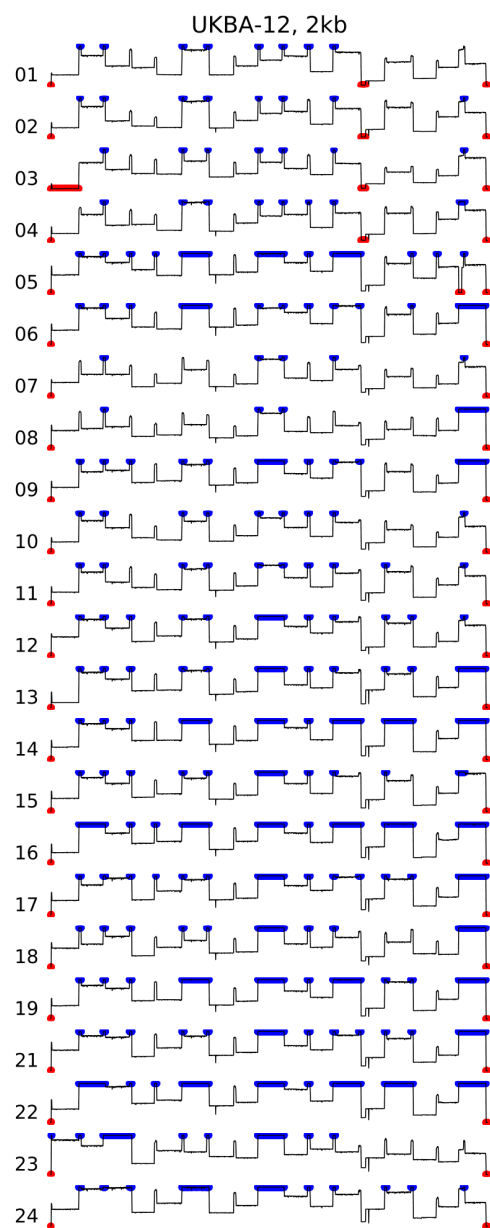

## S2 Fig (continued)

**D**

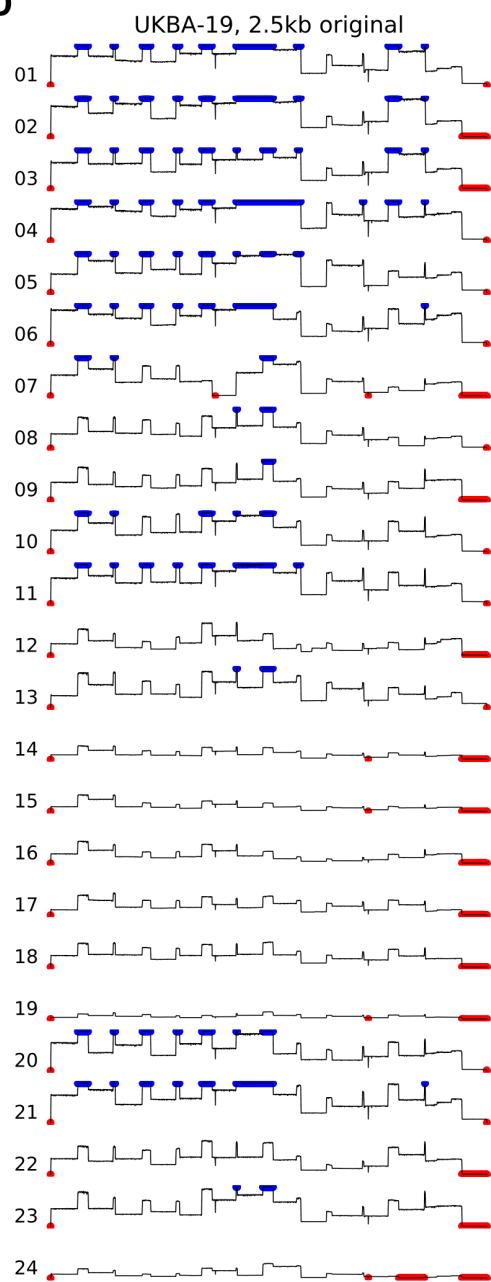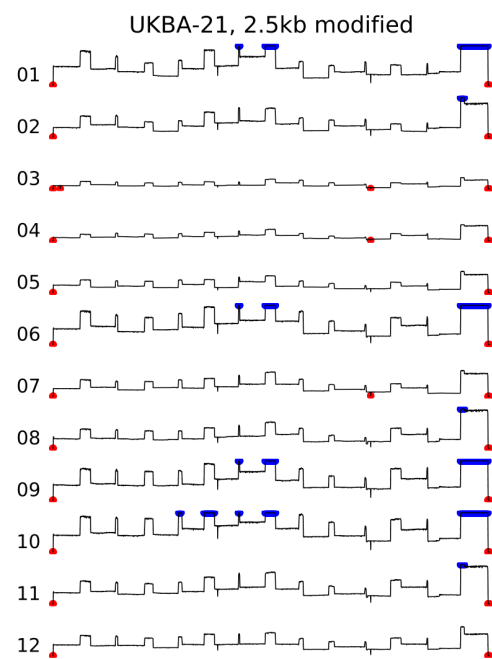

Supplement: S2 Fig — In all runs, an initial portion of the run containing on average 40-Mbp of sequencing data per barcode was used. Coverage values higher than 1000 were clipped at this value and are shown in blue. Coverage below 20 (default Artic cutoff) is shown in red. Medians of 10-bp windows are shown for smoothing. (PDF) [file pone.0259277.s003.pdf]

# S3 Fig

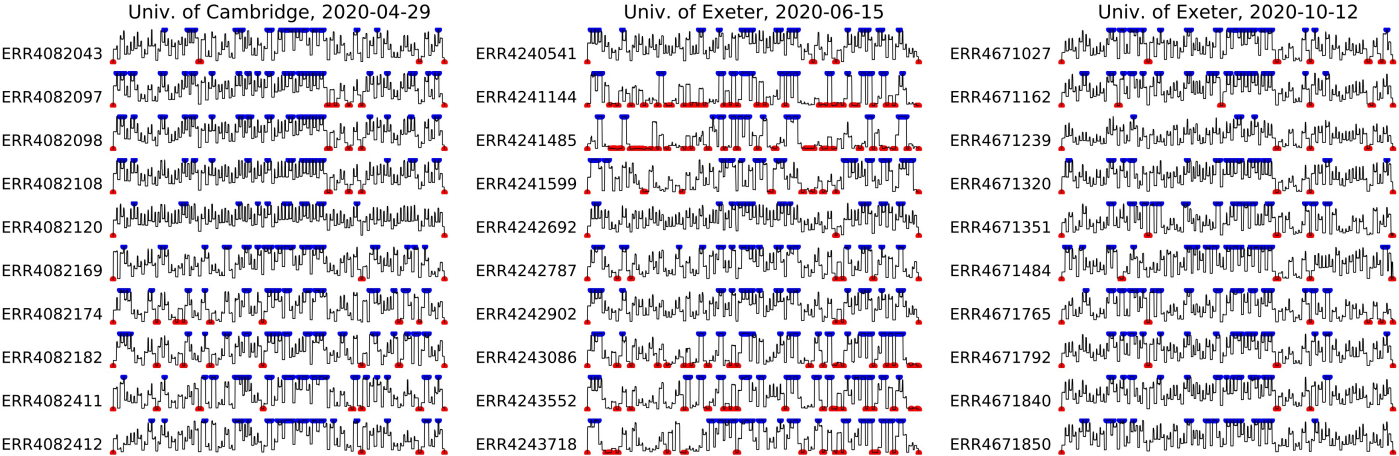

Supplement: S3 Fig — Data by the COVID-19 Genomics UK Consortium were downloaded from ENA archive project PRJEB37886 (https://www.ebi.ac.uk/ena/browser/view/PRJEB37886) on August 4, 2021. Two centers within this project, namely the University of Exeter and the University of Cambridge, submitted a large number of samples amplified with 400-bp primer sets and sequenced by MinION sequencer (828 and 231 samples, respectively). Samples were grouped by submission dates and we randomly selected ten samples from submission dates with a large number of samples. We have sampled 20-Mbp of reads from each sample and aligned them to the reference. The plots show the coverage along the genome as in Fig 4 and S2 Fig. Only 15-Mbp were used for sample ERR4671239 as more data was not available. Note that the downloaded reads are already filtered by barcode, size and are all alignable to the reference. In our 400-bp samples shown in Fig 4 and S2 Fig each barcode has a different amount of data aligned due to differences in the quality of individual samples in the run, but the median is 18-Mbp, which is a value similar to the 20-Mbp cutoff used here. (PDF) [file pone.0259277.s004.pdf]
