# Supplementary material for: Nanopore sequencing of SARS-CoV-2: Comparison of short and long PCR-tiling amplicon protocols
Source: PLoS One. 2021 Oct 29;16(10):e0259277. doi: 10.1371/journal.pone.0259277 (PMC8555800; doi:10.1371/journal.pone.0259277)
Supplement: Attachment — Submitted filename: Responses.pdf [file pone.0259277.s006.pdf]

Journal Requirements:

Please review your reference list to ensure that it is complete and correct. If you have cited papers that have been retracted, please include the rationale for doing so in the manuscript text, or remove these references and replace them with relevant current references. Any changes to the reference list should be mentioned in the rebuttal letter that accompanies your revised manuscript. If you need to cite a retracted article, indicate the article's retracted status in the References list and also include a citation and full reference for the retraction notice.

We checked all references and did not identify any retracted article. Nevertheless, we updated references of two preprints ([6] and [14]) which have since been published in peer-reviewed journals as well as the citation of preprint [8].

Reviewer #3: "Nanopore sequencing of SARS-CoV-2: Comparison of short and long PCR-tiling amplicon protocols" by Brejova et al. describes the comparison of different amplification methods for tiled-amplicon SARS-CoV-2 genome sequencing on the Oxford Nanopore MinION or Flongle platforms. The text is well-written and the data is clearly presented. I am somewhat puzzled by the poor performance of the ARTIC 400 bp amplicons in the authors' hands as this protocol has been used extensively and we routinely achieve much more uniform and complete coverage using this protocol (both on Illumina and ONT platforms). Some comparison to the coverage reported here using standard ARTIC primers and published data from other groups should be provided (possibly in the discussion section), to help the reader understand whether this is expected performance of the ARTIC primers or if their data is an outlier for some reason. Despite this, the authors demonstrate a clear advantage in using longer amplicons for Nanopore sequencing (at least in their hands) and these advantages (along with other perks, such as being able to use fewer primers and making re-balancing of pools easier) are clearly articulated in the manuscript. The authors have done a good job of responding to the previous reviewers' comments and I support publication of this manuscript if this additional point below can be addressed.

Major:
-As stated above, the authors should include a discussion of how their ARTIC 400 bp results compare to the balance and coverage obtained by other groups.

Uneven coverage with ARTIC 400-bp protocol has already been observed by others in literature (e.g. [28, 31]), so this pattern is not specific to our experiments. We have also downloaded several data sets from COG UK member groups and confirmed that similar patterns occur in their data as well (now added as S3 Fig). We now summarize this in the conclusion section of the revised manuscript.

Minor:
-Figure 5. Showing the percent genome coverage (at some threshold like 10x or 20x) would be a more informative metric here as one could still have high average coverage, but low total genome coverage if the reads are skewed to a subset of the amplicons. However, I think it is fine if these plots remain unchanged as the green dots give a sense of what coverage looks like for the less well-represented amplicons.

The proportion of the genome with coverage below threshold 20x can be seen in coverage plots shown in Fig 4 and S2 Fig (red lines). We have referenced both figures in the caption of Fig 5 to make this connection more apparent.
